# A Systematic Review of the Feasibility and Safety of Using Artificial Intelligence to Personalise Interventions for Eating Disorders

**DOI:** 10.1192/bjo.2026.11107

**Published:** 2026-06-30

**Authors:** Minh Chi Nguyen, Athanasios Hassoulas, Geetanjali Grover Dhawan

**Affiliations:** University of Cardiff, Cardiff, United Kingdom

## Abstract

**Aims:**

Eating disorders (ED) are associated with high morbidity, mortality, and relapse, yet access to evidence-based care remains limited. Digital interventions have improved scalability but often lack responsiveness to individual symptom profiles and changing clinical risk. Artificial intelligence (AI) has been proposed as a means of enabling personalised andadaptive interventions. However, the feasibility and safety of AI-enabled approaches in ED care remain unclear. This review evaluated the feasibility and safety of AI-enabled personalised interventions for anorexia nervosa, bulimia nervosa, and binge eating disorder, and explored potential clinical applications and evidence gaps.

**Methods:**

A systematic review was conducted in accordance with PRISMA 2020 guidelines. MEDLINE, APA PsycInfo, and Global Health were searched alongside grey literature sources including ProQuest, OAIster, EthOS, and Overton for studies published between 2020 and 2025. Eligibility criteria were defined using the SPIDER framework to capture quantitative, qualitative, and mixed methods evidence. Included studies examined AI-based approaches such as machine learning, natural language processing, digital phenotyping, or conversational agents used to personalise ED interventions and report feasibility or safety outcomes. Methodological quality was assessed using the Mixed Methods Appraisal Tool, and findings were synthesised narratively.

**Results:**

Eight studies met inclusion criteria across four domains. The evidence suggests that AI-augmented approaches can feasibly support aspects of personalised ED care, although findings remain early and heterogeneous. Machine learning methods showed potential for individualised risk prediction, relapse monitoring, fidelity monitoring in digital therapies, and just-in-time adaptive interventions, with substantial variability in performance and limited generalisability. Sensor-based systems demonstrated promise for low burden, real-time behaviour detection, but were evaluated in very small samples. Conversational agents were generally acceptable and engaging, though safety and autonomy concerns were identified. Clinician and service user studies indicated cautious openness to AI use, alongside ongoing concerns regarding trust, transparency, empathy, and governance.

**Conclusion:**

AI-enabled personalisation for ED is technically feasible and shows promise in enhancing risk monitoring, treatment augmentation, and continuity of care. However, clinical effectiveness and safety remain unestablished. Prospective trials, diverse samples, systematic monitoring of harms, and integration within ethically governed, AI-augmented, clinician-led models are required before routine clinical implementation.

